# Migraine and Complex Regional Pain Syndrome: A Case-Referent Clinical Study

**DOI:** 10.1155/2017/5714673

**Published:** 2017-10-30

**Authors:** Yohannes W. Woldeamanuel, Corinne Cooley, Katharine Foley-Saldena, Robert P. Cowan

**Affiliations:** ^1^Stanford Headache and Facial Pain Program, Department of Neurology and Neurological Sciences, Stanford University School of Medicine, Stanford, CA, USA; ^2^Division of Pain Medicine, Stanford University, Stanford, CA, USA; ^3^Pacific Graduate School of Psychology, Stanford PsyD Consortium, The Palo Alto University, Palo Alto, CA, USA

## Abstract

We studied clinical phenotype differences between migraineurs* with *CRPS (Mig + CRPS) and those without (Mig − CRPS). Mig + CRPS cases and Mig − CRPS referents aged ≥18 years were enrolled. Diagnosis was made in accordance with International Classification of Headache Disorders-3 beta (ICHD-3 beta) for migraine and Budapest Criteria for CRPS. Migraines both with and without aura were included. A total of 70 Mig + CRPS cases (13% males, mean age 48 years) and 80 Mig − CRPS referents (17% males, mean age 51 years) were included. 33% of Mig + CRPS and 38% of Mig − CRPS exhibited episodic migraine (EM) while 66% of Mig + CRPS and 62% of Mig − CRPS had chronic migraine (CM) (OR = 0.98, CI 0.36, 2.67). Median duration of CRPS was 3 years among EM + CRPS and 6 years among CM + CRPS cohort (*p* < 0.02). Mig + CRPS (57%) carried higher psychological and medical comorbidities compared to Mig − CRPS (6%) (OR 16.7, CI 10.2, 23.6). Higher migraine frequency was associated with longer CRPS duration. Migraineurs who developed CRPS had higher prevalence of psychological and medical disorders. Alleviating migraineurs' psychological and medical comorbidities may help lower CRPS occurrence.

## 1. Introduction

Migraine is associated with high levels of disability [1]. Globally, migraine is the most common neurological burden affecting around 1 billion people worldwide with a crude estimate of 14.7% prevalence [1]. The International Classification of Headache Disorders-3 (ICHD-3, beta version) defines migraines as events with at least 5 headache attacks each lasting 4–72 hours with two of the following four characteristics: a unilateral location, pulsating quality, moderate to severe pain intensity, and aggravation by or causing avoidance of routine physical activity and having at least nausea and/or vomiting or photophobia and phonophobia during the headache duration [2]. Based on the frequency of headache attacks, migraine is classified as either episodic or chronic; episodic migraine (EM) is defined with migraineurs having ≤14 headache days per month while chronic migraine (CM) is diagnosed with 15 or more headache days per month for ≥3 months of which 8 or more days meet criteria for migraine [2]. The annual rate of EM-to-CM progression is 2.5% [3–5] while the 2-yearly rate of CM-to-EM remission has been found to be 26% [6, 7]. Some of the modifiable risk factors for EM-to-CM progression include medication overuse [3], obesity [8], hypertension [9], sleep-related problems (insomnia, habitual snoring, sleep bruxism, and daytime sleepiness) [10], psychiatric problems (depression, anxiety, and somatization disorders) [9], specific migraine features (increased headache frequency [11], allodynia [12], nausea [13], and prolonged headache duration [13]), chronic pain disorders [9], and caffeine consumption [14]. Nonmodifiable risk factors include female gender [15, 16], older age groups [5], lower socioeconomic status [5], genetic background [17], stressful life events such as divorce or moving [18], and head and neck trauma [19]. Good compliance with preventive medication, withdrawal from continuous use of analgesics for headache relief, and regular exercise were found to be significant contributors for CM-to-EM remission [20]. Migraineurs who adopt a regular lifestyle behavior of regular sleep/wake times, regular mealtimes, and daily exercise have higher chances of EM than CM [21]. Both EM and CM have overlapping, and distinct clinical and neurobiologic features [7]. Migraine has societal, personal, and economic burden [22]. Around 1–3% of the global population is estimated to suffer from CM [23]. In the United States, migraine accounts for more than $20 billion in direct (e.g., doctor visits, medications) and indirect (e.g., missed work, lost productivity) expenses each year, with economic burden increasing annually [24]. The cost burden of CM is higher than that of EM [25].

Complex regional pain syndrome (CRPS) is a chronic musculoskeletal pain condition characterized by inflammatory and autonomic features localized to a region of the body that is disproportionate to the preceding injury [26]. CRPS type 1 occurs following an injury or immobilization (e.g., frozen shoulder) which did not directly damage peripheral nerves, while CRPS type 2 features a defined peripheral nerve injury [26]. Beyond vasomotor, sudomotor, motor, and trophic changes, CRPS displays qualities of allodynia, hyperesthesia, and hyperalgesia [26]. The level of disability and symptom severity for CRPS is variable, as it may sometimes spontaneously resolve or may also lead to long-term disability [27]. Improvement in work status or disability appears to plateau if pain lasts more than six months [27]. Though the mechanisms for CRPS development are not completely understood, evidence suggests that contributing factors include peripheral and central sensitization [28], autonomic changes [29], inflammatory and immune alterations [30], central nervous system changes [31], and genetic and psychological factors [32]. Researchers have found that psychological factors may not be present at the time of CRPS onset [33], though lower levels of anxiety correlated with lower pain intensity [27]. Another method of classifying CRPS is based on the temperature of the affected skin as “warm” or “cold” [33–35]. Most cases of CRPS are primarily “warm” progressively changing to become “cold” with chronic CRPS [33, 34].

In both CM and CRPS, changes in the central nervous system are being investigated. These neurobiological changes for migraine include functional alterations such as atypical brain responses to sensory stimuli, absence of the normal habituating response between attacks, atypical functional connectivity of sensory processing regions [36], and structural changes such as volumetric changes in gray and white matter [37], and iron deposition [38]. Neurobiological changes among patients with CRPS include maladaptive reorganization in the primary somatosensory cortex [39, 40], alterations in the left posterior hippocampus, and decreased gray matter in the dorsal insula and left orbitofrontal cortex compared to age-matched controls [41].

Both patients with migraine and CRPS report a significant impact on their quality of life to the point of needing bedrest [42] with reduced engagement in social and recreational activities. Elevated levels of disability and pain severity have been associated with psychological factors such as anxiety in patients with CRPS compared to other chronic pain conditions [32], and higher incidences of depression and anxiety are associated with CM [22, 43]. For migraineurs, high levels of muscle tenderness in the cervical and pericranial areas are associated with anxiety and depression [43], suggesting that these psychological comorbidities may have a role in altering pain processing. Psychoeducation on the effect of psychological comorbidities on disease progression and stabilizing psychological comorbidities is important in managing pain symptoms [43].

The main purpose of this study was to describe the features of patients suffering from both migraine and CRPS.

## 2. Methods

### 2.1. Study Question

To describe the features of patients suffering from both migraine and CRPS, we designed a case-referent clinical study examining migraineurs with CRPS (cases) and* without* CRPS (referents).

### 2.2. Study Setting

The study setting was Stanford Headache and Facial Pain Clinic in Stanford, California, a quaternary referral clinical and research center. Patients are provided a wide range of personalized management options through the clinic's multidisciplinary program involving headache medicine, pain medicine, pain psychology, physical therapy, psychophysiological therapy (for, e.g., biofeedback), and integrative medicine (for, e.g., acupuncture, hypnosis).

### 2.3. Enrollment and Ethical Approval

Enrollment was conducted from a cohort population identified using the Stanford Translational Research Integrated Database Environment (STRIDE) [44] Clinical Data Review Tool based on electronic medical records seen and followed at the Stanford Headache and Facial Pain Clinic. STRIDE [44] is a clinical informatics research and development project at Stanford University to create a standards-based informatics platform supporting clinical and translational research. Ethical clearance from the Stanford University IRB (Institutional Review Board) was sought and full approval was obtained. Methods and design of the study were in accordance with STROBE (STrengthening the Reporting of OBservational studies in Epidemiology) [45] checklist.

### 2.4. Inclusion and Exclusion Criteria

Clinical documents recorded from January 1, 2014, until January 1, 2016, were screened for possible inclusion and exclusion. All patients aged 18 years and older diagnosed with both migraine and CRPS were included as* cases *(Mig + CRPS). In order to match the cases, a convenience sample of migraine patients* without* CRPS aged 18 years and older was included as* referents* (Mig − CRPS). Patients aged younger than 18 years were excluded.

### 2.5. Data Abstraction

Data was abstracted by two investigators (YWW and CC). Interrater reliability (IRR) was assessed utilizing Cohen's *κ*. Data abstractors were not blinded from the study objective. The following data were extracted from each patient: age, gender, marital status, type of migraine (episodic migraine, chronic migraine, migraine with aura, and migraine without aura), age at first migraine attack, type of CRPS (CRPS type 1 or type 2), age at CRPS onset, migraine medications, CRPS medications, psychological comorbidity, physical therapy and disability, diagnostic method, medical comorbidity, initial and current headache status, and initial and current CRPS status. Missing data was excluded from the analysis.

### 2.6. Sample Size: Case-Referent Power Analysis

Assuming a 25% and 50% proportion of hypothetical exposure (for, e.g., psychological comorbidity) among the referents and cases, respectively, with a 1 : 1 case-to-referent ratio and two-sided confidence level of 95%, a sample size of 60 to 90 patients in each cohort achieves 80% −85% power to detect a minimum OR of 2-3. Hence, a 2-year timespan of our study period involving all Mig + CRPS cases that fulfilled our inclusion criteria yielded 70 cases; 80 patients with Mig − CRPS were included as referents. This rendered a final sample size of 150 patients. OpenEpi, Version 3 was utilized to compute sample size and power analysis [46].

### 2.7. Statistical Analyses

D'Agostino & Pearson omnibus normality test was applied prior to selecting parametric tests. Other appropriately employed tests include* t*-tests, one-way ANOVA, Mann–Whitney test, Kruskal-Wallis, and Dunn's post hoc test. Odds ratio (OR) with 95% Confidence Interval (CI) was used to measure associations between exposures and outcomes. Statistical significance was set at the level of 0.05.

## 3. Results

**Figure 1:**
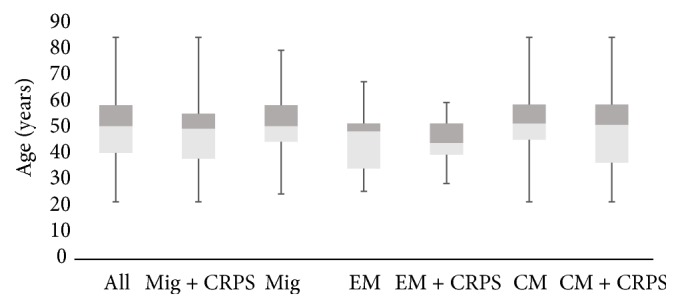
*Age distribution among the different cohorts.* Age distribution was normally distributed among all cohorts (D'Agostino & Pearson omnibus normality test). There was no statistically significant intermean difference in age among the different cohorts (*p* = 0.09, ANOVA). The following order was noted in increasing order (mean age in years): EM + CRPS (45), EM (46), Mig + CRPS (48), CM + CRPS (49), All (49.5), Mig (51), and CM (52). Migraine cohorts with CRPS (Mig + CRPS, EM + CRPS, CM + CRPS) were observably younger than their counterparts* without* CRPS (Mig − CRPS, EM − CRPS, CM − CRPS). Means, interquartile ranges, minimum, and maximum values are displayed by boxes and whiskers.

Diagnosis was made clinically and in accordance with the International Classification of Headache Disorders-3 beta (ICHD-3 beta) [2] for migraine and the Budapest Criteria for CRPS. A total of 70 cases (14% males) having both migraine and CRPS (Mig + CRPS) were included in the final analysis. Cohen's for IRR was 0.85. Age distribution was parametric according to D'Agostino & Pearson omnibus normality test (*p* = 0.32) with a mean of 48 years (SD 13) (Figure 1). Additional 80 referent cases (16% males) having migraine only (migraine* without* CRPS or Mig − CRPS) were included from patients consecutively seen at the clinic within the same year; D'Agostino & Pearson omnibus normality test (*p* = 0.98) revealed parametric distribution with a mean of 51 years (SD 13) among the referents (Figure 1). There was no statistically significant intermean difference in age among the different cohorts (*p* = 0.09, ANOVA); however the following order was noted in increasing order (mean age in years): EM + CRPS (45), EM (46), Mig + CRPS (48), CM + CRPS (49), All (49.5), Mig (51), and CM (52) (Figure 1).

Sex differences between the two cohorts were similar and were not associated with CRPS occurrence (odds Ratio of 1.30, 95% CI 0.33, 5.11). Half of the Mig + CRPS patients were single/divorced (50%) compared to the Mig − CRPS cohort (37%) (odds Ratio of 0.67, 95% CI 0.21, 2.16); the rest were married. Information on level of education was not available in the majority of the cases. Family history of headache was present in 71% of the Mig + CRPS cohort.

**Figure 2:**
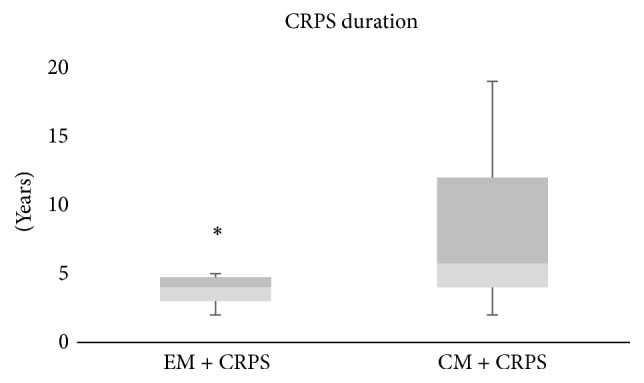
Duration of CRPS among the two cohorts of EM + CRPS (episodic migraineurs with CRPS) and CM + CRPS (chronic migraineurs with CRPS). CRPS duration was nonparametrically distributed with a median of 3 years (IQR 3–5) among the EM + CRPS cohort and 6 years (IQR 4–12) among the CM + CRPS cohort (Mann–Whitney test, *p* < 0.02). Medians, interquartile ranges, minimums, and maximums are displayed with boxes and whiskers. *∗* represents *p* value < 0.05.

Thirty-three% of the Mig + CRPS cohort exhibited EM while the remaining 66% had CM. Similarly, 38% of the Mig − CRPS cohort exhibited EM while the remaining 62% suffered from CM. The frequency of migraine days was not significantly associated with CRPS occurrence (odds Ratio of 0.98, 95% CI 0.36, 2.67). Age at first migraine attack was around early school-age and was found to be comparable between the two cohorts of Mig + CRPS and Mig − CRPS. Migraine onset preceded CRPS onset in all cases of Mig + CRPS with a median of 18.5 years (IQR 10–25). Duration of CRPS was nonparametrically distributed with a median of 3 years (IQR 3–5) among the EM + CRPS (Episodic Migraineurs with CRPS) cohort and 6 years (IQR 4–12) among the CM + CRPS (Chronic Migrainuers with CRPS) cohort (Mann–Whitney test, *p* < 0.02; Figure 2). EM + CRPS cohort exhibited observably higher prevalence of CRPS type 2 (60%) compared to CM + CRPS cohort (12%) (odds Ratio of 0.22, 95% CI 0.03, 1.73). Two (7%) of the Mig + CRPS patients had migraine with aura, while all Mig − CRPS had migraine without aura.

**Figure 3:**
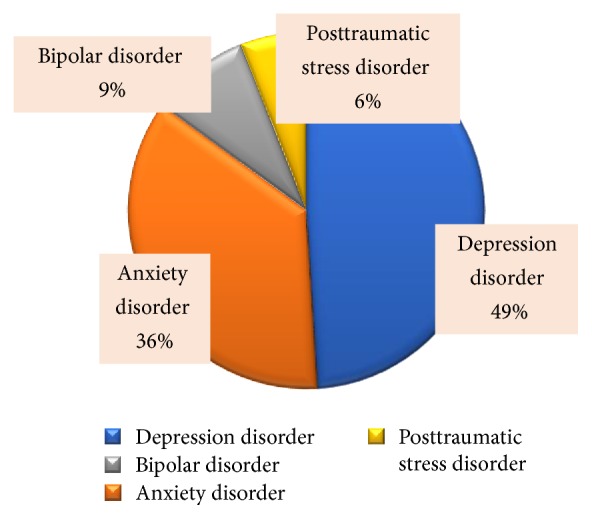
*Psychological comorbidities among all patients.* Past or present psychological comorbidities included depression disorder (49%), anxiety disorder (36%), bipolar disorder (9%), and posttraumatic stress disorder (6%).

**Figure 4:**
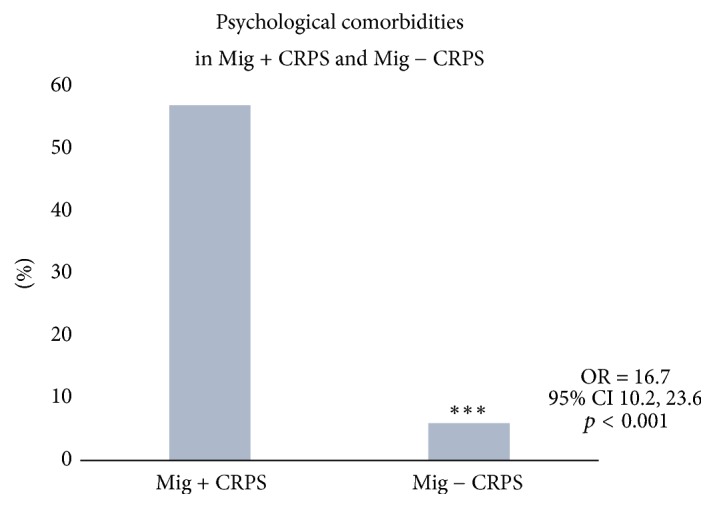
*Comparison between the cohorts of Mig + CRPS (migraine with CRPS) and Mig − CRPS (migraine without CRPS).* Mig + CRPS (57%) cohort exhibited higher burden of psychological problems compared to the Mig − CRPS cohort (6%) (OR 16.7, 95% CI 10.2, 23.6; *p* = 0.0002). *∗∗∗* represents* p *value < 0.001.

Triggers for CRPS included fall injuries (41%), postsurgical wounds (41%), fractures (8%), motor vehicle accidents (5%), idiopathic causes (3%), and others (3%). Among all 150 patients, past or present psychological comorbidities included depression disorder (49%), anxiety disorder (36%), bipolar disorder (9%), and posttraumatic stress disorder (6%) (Figure 3). Mig + CRPS (57%) cohort exhibited higher burden of psychological problems compared to the Mig − CRPS cohort (6%) (OR 16.7, 95% CI 10.2, 23.6; *p* = 0.0002). Past or current medical comorbidities were also more common among the Mig + CRPS cohort compared to Mig − CRPS cohort. Sixteen% of the Mig + CRPS cohort had history of previous opioid-containing pain medication use to manage their headache compared to 3% of the Mig − CRPS cohort (OR = 4.33, 95% CI 3.64, 6.07) (Figure 4). Butalbital-containing medications were used by same percentage of cases (3%) among both the Mig + CRPS and Mig − CRPS cohorts. Patients overusing opioids and butalbital-containing medications were referred for multimodal detoxification treatment. Overall missing data was 5% in Mig + CRPS cohort and 4% in Mig − CRPS cohort.

## 4. Discussion

Migraine is significantly associated with CRPS [33, 47–49], prompting some authors to call migraine as CRPS of the brain [47, 50] whereas other experts coined the term* “migrainous corpalgia”* to suggest a shared pathophysiology of central sensitization between the two conditions while describing migraine's cephalic and extracephalic cutaneous allodynic presentations [51]. Migraine and CRPS have shared pathogenetic backgrounds [33, 52–56]. Neuropeptides such as CGRP [52, 54], mast cells [53], and neurogenic inflammation [52, 54, 55] and reactive oxygen species (ROS) [56] are involved in both migraine and CRPS. Our study probed further into the comparison of clinical phenotypes of migraineurs who developed CRPS (cases or Mig + CRPS) and those who did not (referents or Mig − CRPS).

In this study, sociodemographic characteristics of both cases and referents displayed middle aged adults with females being affected 4 times more often than males. Both migraine and CRPS are known to have peak incidence in age groups of 30–50, featuring female preponderance; migraine affects females twice as often as males [57, 58] while CRPS affects females 4 times more commonly [59]. Our study revealed that migraine cohorts with CRPS (Mig + CRPS, EM + CRPS, and CM + CRPS) were observably younger than their counterparts* without* CRPS (Mig − CRPS, EM − CRPS, and CM − CRPS); this indicated that CRPS might be more common in younger patients. In another study, age of onset of CRPS cases with migraine (35 years) was reported to be a decade earlier than those* without* migraine (47 years) [49]. Similarly, cross-sectional studies have found that both EM and CM patients with cutaneous allodynia were up to 6 years younger than those without [60, 61]. Future studies need to explore explanations for this difference in detail and study the impact of headache chronicity: could this be because florid autonomic symptoms are more common accompaniments in younger migraine and CRPS patients? Could a younger autonomic system feature stronger autonomic responses in these conditions?

Marital status was not significantly different between our two cohorts of Mig + CRPS and Mig − CRPS; this was similar to results from another study [49]. Our sampling method involved convenience sampling of consecutive cases and referents seen at the clinic in two-year period. While convenience sampling is the most common and efficient method of sampling, it is known to lead to unintentional nonrandom selection biases [62, 63]. However, the cases and referents included in our study had similar baseline sociodemographic characteristics compared to the overall migraine and CRPS epidemiology, and this counterbalances the potential for sampling-related selection bias. CRPS occurrence was similar among both males and females with migraine. Although migraineurs who were married observably seemed to be protected against CRPS, this was not found to be statistically significant. High level of IRR in our study implied the data points of this study were consistent among data abstractors.

CM was nearly twice as common as EM among both cases and referents; this could be because a referral center receives higher number of patients with CM compared to EM. Both episodic and chronic migraineurs were found to have similar chances of developing CRPS. This indicated that frequency of migraine attacks was not related to CRPS progression.

Age of first migraine attack was in childhood. This is similar to the reported epidemiology of first migraine attack which is also in childhood [64–66]. More than 80% of migraineurs have their first attack by the age of 30 [67]. In our study, there was a latency period of approximately 2 decades between first migraine attack and onset of CRPS. Postdiagnosis median duration of CRPS among our CM cohort (6 years) was twice as long as that among the EM cohort (3 years). Another study exploring CRPS and migraine association found mean disease duration of CRPS to be about 9 years [49]. Other studies showed delayed diagnosis of CRPS for up to 20 years from symptom onset; a median diagnosis delay of 6 months and 1 year has been reported in the UK [68] and the Netherlands [69], respectively. Diagnostic delay for migraine has been reported to be up to 12 years [34, 70]. While migraine is classified as a chronic disorder with episodic attacks, CRPS is largely monophasic with only 2% cases reported as relapsing-remitting [69, 71].

With regard to the prognosis of CRPS, there are mixed results worldwide. A population-based study in the US indicated that 70–80% of new incident CRPS cases had achieved complete and spontaneous relief of their symptoms within a period of 1 year [59, 72]; these results were based on medical chart reviews. However, other European prospective studies ranging from 3 to 9 years revealed higher disability and longer persistence [69, 73, 74], one study reporting complete remission in 30% and stabilizing course in 54%. The CRPS cases included within our study are those with longstanding symptoms; this may be due to the referral nature of our clinical setting where more disabling cases are seen. Our results relate to the findings by other studies which indicate that migraine association with CRPS occurs among the chronic and longstanding CRPS patients known as “cold” type of CRPS which account for about 30% of all CRPS presentation [33, 74]. “Cold” CRPS manifests with higher levels of central sensitization, disease progression, and poor pain outcomes [33, 74]. In our study, we found higher frequency of CRPS type 2 among the Mig + CRPS cases. There are no studies in the medical literature exploring which CRPS type is associated with migraine; we suggest future research addressing this area. There is currently an ongoing long-term prospective registry of CRPS cases for 15 years; results from this data can provide clues into the disease progression and accompanying comorbidity of migraine [68].

Higher occurrence of aura among our Mig + CRPS cohort was also observed in another study [49]; similarly, migraine patients with allodynia were found to have higher incidence of aura [61].

Antioxidative supplements such as vitamin C have been found to be efficacious in CRPS, while, in migraine, vitamin E has been shown to be efficacious [35]. In this regard, exploring the role of vitamin C for migraine management has utility [50].

The majority of our Mig + CRPS cohort had first-degree family history of headache. First-degree family history of headache was found to be a risk factor for CRPS occurrence and hence prophylactic treatment during surgical procedures should be considered for migraine patients with higher levels of extracephalic allodynia, so as to prevent CRPS progression.

In our study, there were more psychological comorbidities among the Mig + CRPS cohort compared to the Mig − CRPS cohort; this was consistent with previous work [49]. Undertreating these comorbidities may have huge implications for disability and can have a negative impact on patients' quality of life. These psychological comorbidities, such as anxiety, as stated earlier have been correlated with pain intensity in CRPS. A comprehensive pain management plan of care for patients with both Mig + CRPS should address these psychological comorbidities, in addition to retraining their altered sensory processing through rehabilitation techniques such as Graded Motor Imagery and medication management [75, 76].

Limitations of our study are inherent to retrospective designs: consecutive sampling and abstractors not being masked. We plan to conduct a prospective study with larger sample size to power results from possible confounding variables. However, the rarity of CRPS has to be factored which was why we employed a case-referent study. That the sociodemographics of the cohorts in our study are similar to that seen in real world cases and community-based studies supports the fact that our results can have external validity, replicability, and generalizable across different settings. However, additional studies with similar topic can strengthen the generalizability of our results.
